# Serum levels of cytokines in infants treated with conbercept for retinopathy of prematurity

**DOI:** 10.1038/s41598-020-69684-7

**Published:** 2020-07-29

**Authors:** Yong Cheng, Xuemei Zhu, Dandan Linghu, Yongsheng Xu, Jianhong Liang

**Affiliations:** 1 Department of Ophthalmology and Clinical Centre of Optometry, Peking University People’s Hospital, Eye Diseases and Optometry Institute, Beijing Key Laboratory of Diagnosis and Therapy of Retinal and Choroid Diseases, College of Optometry, Peking University Health Science Center, 11 Xizhimen South Street, Xicheng District, Beijing, 100044 China; 2 Clinical Stem Cell Research Center, Peking University Third Hospital, 49 Huayuan North Road, Haidian District, Beijing, China

**Keywords:** Paediatric research, Clinical trial design, Drug therapy, Adverse effects

## Abstract

Intravitreal anti-vascular endothelial growth factor (VEGF) agents have revolutionized the treatment of retinopathy of prematurity (ROP); however, there are concerns regarding the potential systemic complications caused by those treatments. This study aimed to determine the serum concentrations of cytokines in infants with ROP and to evaluate the changes in serum VEGF concentrations after intravitreal conbercept (IVC). Sixty infants with ROP treated with IVC 0.25 mg were included. Blood samples were collected before treatment as well as 1 week and 4 weeks after treatment. Serum levels of 45 types of cytokines were measured by a multiplex bead assay. We observed that IVC 0.25 mg in ROP patients suppressed the circulating levels of VEGF-A and VEGF-D as of 1 week after injection, and these growth factor levels returned to baseline at 4 weeks. No significant differences were observed in the serum levels of the other cytokines between baseline and 1 or 4 weeks after IVC.

## Introduction

Retinopathy of prematurity (ROP) is a multifactorial retinal disease that remains a major treatable cause of childhood blindness worldwide^1^. Especially in developing countries, the prevalence of ROP is growing considerably due to the increased survival rate of low-body-weight preterm infants. Vascular endothelial growth factor (VEGF) is a key proangiogenic factor involved in the pathogenesis of ROP, promoting retinal neovascularization and prompting researchers to explore the effect of anti-VEGF agents in the management of ROP. Recently, the use of anti-VEGF drugs has revolutionized the treatment of ROP, as these agents have several distinct advantages over conventional standard laser photocoagulation therapy, including a simpler procedure and reduced risks of myopia and peripheral visual field defects^2^. However, given the potential for local and systemic adverse effects from these drugs, there are concerns regarding their use. A few studies have indicated that intravitreal anti-VEGF drugs induce systemic VEGF suppression for weeks to months^3–5^, although no significant adverse events have been reported to date. There have been occasional reports on the use of conbercept, a novel anti-VEGF agent, for ROP treatment^6–8^, however, the effect of conbercept on systemic VEGF concentrations in ROP has not yet been reported.

In addition to angiogenesis, an increasing number of studies have demonstrated that inflammation might play an important role in the pathogenesis of ROP. Epidemiological studies have observed elevated rates of ROP in patients with perinatal infections or inflammation, indicating that inflammation may disturb the development of retinal vessels^9,10^, this observation has been confirmed in animal models^11^. In addition, eight cytokines were detected to be significantly different in ROP patients in different time periods^12^. Serum levels of interleukin (IL)-7, monocyte chemotactic protein-1 (MCP-1), macrophage inflammatory protein 1 alpha (MIP-1α), and MIP-1β were significantly elevated in preterm neonates who went on to develop ROP^13^. In another study, plasma levels of IL-6, IL-8, and tumor necrosis factor-α (TNF-α) were increased in ROP patients^14^. Holm et al. reported that preterm infants with systemic inflammation in the first postnatal month had an increased risk of prethreshold ROP^15^. Thus, further studies are required to obtain a better understanding of the molecular mechanisms involved in ROP, guide treatment and identify predictors of ROP.

The aim of this study was to assess the changes in serum levels of VEGF and other cytokines in ROP infants who received intravitreal conbercept (IVC). To our knowledge, this is the first study to present the effect of conbercept on systemic VEGF concentration in ROP patients.

## Methods

This study was a case series study approved by the Ethical Committee and Institutional Review Board of Peking University People’s Hospital (Beijing, China). Written informed consent was obtained from the guardian of each participant in accordance with the Declaration of Helsinki. All the examinations, diagnoses and treatments of participants were performed at the Ophthalmology Department of Peking University People’s Hospital. Sixty preterm infants who were diagnosed with ROP and received an intravitreal injection of 0.25 mg conbercept from November 2017 to September 2018 were enrolled in the study group, while the control group consisted of 10 infants diagnosed with congenital cataract.

In the ROP group, we communicated with the participants’ parents regarding the off-label use of IVC and the potential adverse effects of the procedure before initiating treatment. ROP was classified on the basis of the International Classification of Retinopathy of Prematurity (ICROP) as updated in 2005^16^ and was diagnosed by experienced pediatric ophthalmologists (Y.C. and J.L.). The inclusion criteria for the ROP group included (i) a diagnosis of Zone I/II Stage 2/3 ROP with plus disease or aggressive posterior ROP (AP-ROP) with no previous treatment,(ii) the absence of major congenital anomalies,and (iii) no blood transfusion in the 2 weeks preceding blood collection.

Venous blood samples from ROP patients were collected in plastic serum separator tubes before IVC as well as 1 week and 4 weeks after IVC, while those of the control group were collected at the time of diagnosis. Samples were then centrifuged at 3,000 rpm for 10 min, and the supernatants were stored in Eppendorf tubes at − 80 °C until assayed. A 45-plex Human Cytokine/Chemokine/Growth Factor Procarta Plex Panel (cat. # EPXR450-12171-901, Invitrogen, Thermo Fisher Scientific) was used to measure the serum concentrations of cytokines via Luminex technology as described previously^17^^,^ including VEGF-A, VEGF-D, placental growth factor-1 (PlGF-1), platelet-derived growth factor-BB (PDGF-BB), brain-derived neurotrophic factor (BDNF), β-nerve growth factor (β-NGF), epidermal growth factor (EGF), basic fibroblast growth factor (FGF-2), granulocytemacrophage colony stimulating factor (GM-CSF), hepatocyte growth factor (HGF), stem cell factor (SCF), stromal cell derived factor-1α (SDF1α), eotaxin, growth-regulated oncogene α (GROα), interferon gamma (IFN-γ), IFNα, TNF-α, TNF-β, IL-1RA, IL-1β, IL-1α, IL-2, IL-4, IL-5, IL-6, IL-7, IL-8, IL-9, IL-10, IL-12 p70, IL-13, IL-15, IL-17A, IL-18, IL-21, IL-22, IL-23, IL-27, IL-31, interferon-induced protein10 (IP-10), leukemia inhibitory factor (LIF), MCP-1, MIP-1α, MIP-1β, and regulated upon activation normal T cell (RANTES). All measurements were performed in triplicate.

Data are presented as the mean ± standard deviation and were analyzed using SPSS 20.0 for Mac (SPSS, IBM Corp., NY, USA). The Mann–Whitney U test was conducted to compare differences between ROP patients and controls. The Wilcoxon matched-pairs signed rank test was used to compare the cytokine changes in ROP patients before and after IVC injection. A *P* value less than 0.05 was considered statistically significant.

## Results

### Clinical data and treatment outcomes of the study population

Sixty patients (27 girls and 33 boys, 117 eyes) with Zone I/II Stage 2/3 ROP with plus disease or AP-ROP were enrolled in the study, and 10 full-term infants (four girls and six boys, 15 eyes) with congenital cataracts were included as the control group. The demographics of the patients are summarized in Table 1. The mean gestational age of infants with ROP was 29.02 ± 1.67 weeks (range 26–34.71 weeks), and their mean birth weight was 1,173.13 ± 316.50 g (range 500–2,100 g). The mean postmenstrual age at initial treatment was 39.70 ± 3.32 weeks (range 33.29–51 weeks). The mean age of the control group was 4.95 ± 2.15 months. Recurrence that required treatment occurred in 9 patients (15 eyes, 12.82%) with ROP, and the mean recurrence interval was 6.28 ± 5.41 weeks (range 2 to 20.5 weeks). The remaining eyes exhibited regression of the disease after one injection. Repeated intravitreal anti-VEGF agent injection or laser photocoagulation was performed for the eyes with recurrence. All patients with ROP were followed up for at least 6 months; the mean length of follow-up was 48.34 ± 11.46 weeks. At the end of the follow-up, no ocular or systemic adverse events were observed in this group, and all the eyes achieved favorable treatment outcomes, with no signs of recurrence or retinal detachment.

**Table 1:** Demographicsof study population.

Characteristic		ROP patients	Controls	*P* value
No		60 (117 eyes)	10 (15 eyes)	
Female, no. (%)		27 (45%)	4 (40%)	0.77^a^
Age		39.70 ± 3.32 (weeks)	4.95 ± 2.15 (months)	< 0.001^b^
PMA at treatment (weeks)		39.70 ± 3.32 (33.29–51)	/	
GA (weeks)		29.02 ± 1.67 (26–34.71)	/	
Birth weight (g)		1,173.13 ± 316.50 (500–2,100)	/	
Zone, Stage, no. (eyes)			/	/
I 3 +		3		
II 2 +		13		
II 3 +		93		
AP-ROP		8		
Recurrence		9 (15 eyes)	/	/
Recurrence interval (weeks)		6.28 ± 5.41 (2.0–20.5)	/	/
Follow-up periods (weeks)		48.34 ± 11.46	/	/

Data are shown as mean ± SD or number (%).

*ROP* retinopathy of prematurity, *PMA* postmenstrual age, *GA* gestational age, *AP-ROP* aggressive posteriorretinopathy of prematurity.

^a^X2 test.

^b^Mann–Whitney U testwas performed to compare age between two groups.

### Changes in serum levels of cytokines in ROP patients before and after treatment with conbercept

**Figure 1:**
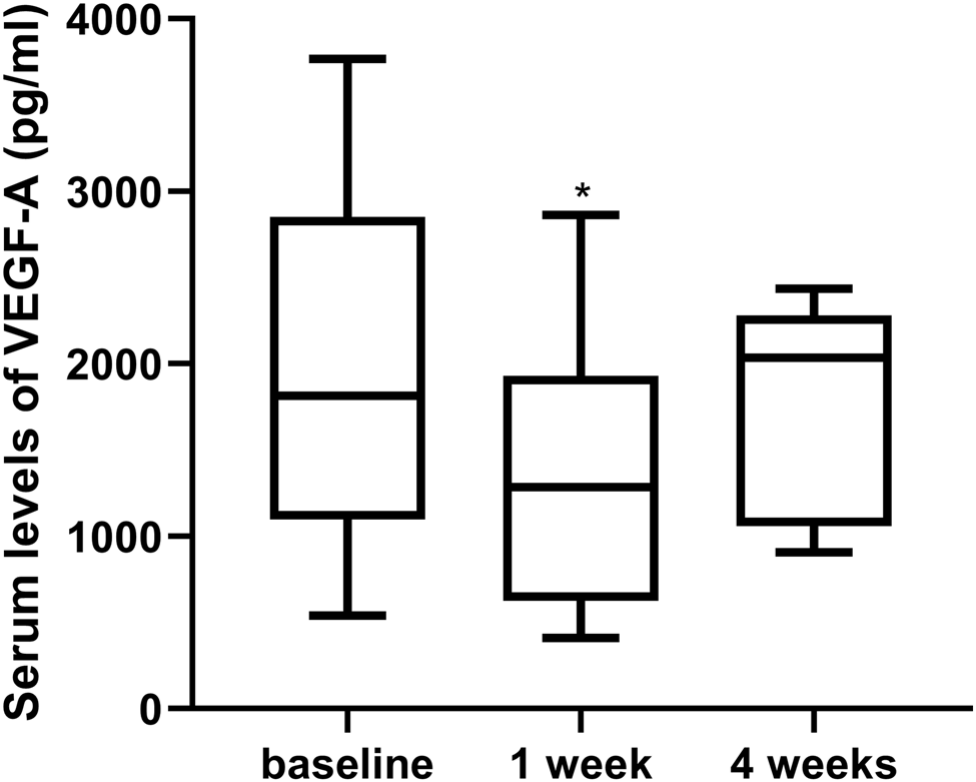
Box plot showing the serum VEGF-A levels of ROP patients before and after IVC. The serum VEGF-A levels at 1 week after IVC were significantly lower than baseline. No significant difference was observed between baseline and 4 weeks after IVC. *P < 0.05.

The mean serum levels of VEGF-A before, 1 week after, and 4 weeks after an injection of 0.25 mg conbercept were 1976.18 ± 982.45 pg/ml (n = 60), 1,388.99 ± 753.27 pg/ml (n = 16), and 1771.53 ± 581.78 pg/ml (n = 8), respectively. The serum VEGF-A level at 1 week after IVC was significantly lower than baseline (*P* = 0.021). However, no significant difference was detected between baseline and 4 weeks after IVC (*P* = 0.25) (Fig. 1).

**Figure 2:**
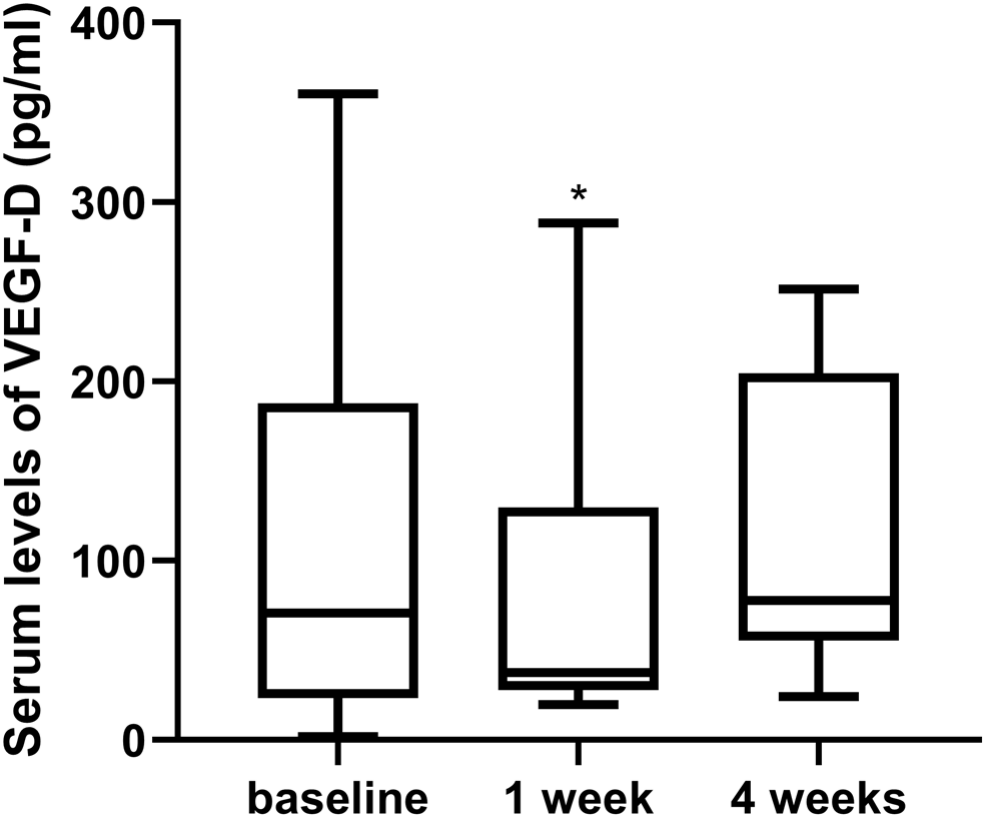
Box plot showing the serum VEGF-D levels of ROP patients before and after IVC. The serum VEGF-D levels at 1 week after IVC were significantly lower than baseline. No significant difference was observed between baseline and 4 weeks after IVC. *P < 0.05.

The mean serum levels of VEGF-D before, 1 week after, and 4 weeks after an injection of 0.25 mg conbercept were 110.23 ± 99.57 pg/ml (n = 60), 87.88 ± 90.34 pg/ml (n = 16), and 111.06 ± 79.99 pg/ml (n = 8), respectively. The serum VEGF-D level at 1 week after IVC was significantly lower than baseline (*P* = 0.044). However, no significant difference was detected between baseline and 4 weeks after IVC (*P* = 0.46) (Fig. 2).

In this study, 21 cytokines (GM-CSF, IL-1α, IL-1β, IL-2, IL-4, IL-5, IL-7, IL-9, IL-10, IL-12 p70, IL-13, IL-15, IL-17A, IL-21, IL-22, IL-23, IL-27, IL-31, TNF-β, LIF, and IFN-α) had concentrations lower than the minimum detectable levels in more than 50% of the samples. No significant differences were observed in serum levels of the other 22 cytokines between baseline and 1 week or 4 weeks after IVC injection (Supplemental Table 1).

### Serum levels of cytokines in infants with ROP

Compared with the control group, patients with ROP had increased levels of the cytokines VEGF-A (*P* = 0.002), VEGF-D (*P* = 0.012), MIP-1β (*P* = 0.009), IP-10 (*P* = 0.008), RANTES (*P* = 0.002), eotaxin (*P* = 0.017), TNF-α (*P* = 0.019), IL-18 (*P* = 0.025), EGF (*P* < 0.001), SCF (*P* = 0.007), PlGF-1 (*P* = 0.008), and β-NGF (*P* = 0.034), along with decreased levels of MCP-1 and HGF (*P* < 0.001 and *P* < 0.001, respectively). No significant difference was observed in the concentration of MIP-1α, IL-6, IL-8, SDF-1α, GRO-α, IFN-γ, IL-1RA, FGF-2, PDGF-BB, and BDNF between the two groups. Detailed results are presented in Table 2. The other 21 cytokines mentioned above were not analyzed.

**Table 2:** Serum concentrations (pg/ml) (mean ± SD) of cytokines in ROP patients (study group) and subjects with congenital cataract (control group).

Cytokines	ROP patients	Controls	*P* value
VEGF-A	1976.18 ± 982.45	683.0 ± 452.8	0.002
VEGF-D	110.23 ± 99.57	26.1 ± 48.1	0.012
MIP-1β	194.64 ± 146.67	66.0 ± 74.3	0.009
Eotaxin	29.72 ± 24.13	10.6 ± 9.3	0.017
IP-10	136.99 ± 111.45	37.9 ± 29.2	0.008
RANTES	27.50 ± 17.30	9.1 ± 11.8	0.002
IL-18	48.97 ± 41.92	16.8 ± 32.5	0.025
TNF-α	14.30 ± 8.10	7.8 ± 5.6	0.019
MCP-1	232.96 ± 255.37	560.6 ± 280.7	< 0.001
EGF	50.31 ± 39.13	2.6 ± 4.2	< 0.001
SCF	23.50 ± 15.91	9.3 ± 2.6	0.007
PlGF-1	45.62 ± 37.48	11.3 ± 21.7	0.008
β-NGF	22.31 ± 16.00	10.7 ± 10.4	0.034
HGF	113.47 ± 113.59	678.0 ± 419.3	< 0.001
SDF-1α	268.55 ± 204.30	221.6 ± 56.7	0.48
GRO-α	28.26 ± 35.30	7.1 ± 2.4	0.07
BDNF	62.75 ± 78.73	12.6 ± 16.1	0.05
IFN-γ	58.74 ± 59.31	19.2 ± 43.3	0.05
IL-8	16.32 ± 29.74	14.2 ± 8.4	0.83
IL-6	14.31 ± 10.50	24.3 ± 33.9	0.12
MIP-1α	17.26 ± 23.20	10.7 ± 3.5	0.39
IL-1RA	892.19 ± 1,388.95	585.1 ± 257.8	0.49
FGF-2	16.63 ± 8.73	17.7 ± 6.9	0.71
PDGF-BB	243.53 ± 388.90	23.0 ± 41.3	0.08

Data are shown as mean ± SD. Mann–Whitney U test.

*ROP* retinopathy of prematurity, *VEGF* vascular endothelial growth factor, *MIP* macrophage inflammatory protein, *IP-10* interferon-induced protein10, *RANTES* regulated upon activation normal T cell, *IL* interleukin, *TNF-α* tumor necrosis factor-alpha, *MCP* monocyte chemotactic and activatingfactor, *EGF* epidermal growth factor, *SCF* stem cell factor, *PIGF-1* placental growth factor-1, *NGF* nerve growth factor, *HGF* hepatocyte growth factor, *SDF1α* stromal cell derived factor-1α, *GROα* growth-regulated oncogene α, *BDNF* brain-derived neurotrophic factor, *IFN-γ* interferon gamma, *IL-1Ra* interleukin-1 receptor antagonist, *FGF-2* basic fibroblast growth factor, *PDGF-BB* platelet-derived growth factor-BB.

## Discussion

In this study, we observed that 1) serum VEGF-A and VEGF-D levels were suppressed at 1 week after IVC and returned to baseline at 4 weeks, while no significant differences were observed in the serum levels of other cytokines between baseline and 1 week or 4 weeks after IVC injection; 2) compared with the control group, ROP patients had significantly different serum concentrations of several angiogenic, inflammatory, and chemotactic factors, indicating that those factors may be associated with ROP.

The main concerns of anti-VEGF therapies, a set of recently emerged treatments for ROP recently, are potential side effects of systemic VEGF suppression. A few studies have indicated that intravitreal anti-VEGF drugs induce systemic VEGF suppression for weeks to months. Sato et al.^3^ reported that serum VEGF levels significantly decreased 1 week after 0.5 mg intravitreal bevacizumab (IVB) in 4 patients. Wu et al.^18^ found that serum VEGF levels were suppressed at 1 day after IVB in 3 patients and still measurable in 1 patient at 8 weeks. In a later study by the same group, serum VEGF levels significantly decreased from baseline over a follow-up period of up to 8 weeks in 6 patients with ROP after IVB, while there was no significant difference in 4 patients who underwent intravitreal ranibizumab (IVR) treatment^5^. Hong et al.^19^ found that plasma VEGF was significantly reduced at 1 week and 2 weeks after IVB in 6 patients and returned to baseline at 8 weeks. Our previous study demonstrated that plasma VEGF was suppressed 1 day after IVR and normalized 1 week after injection^20^. Recently, Chen et al.^21^ found that serum VEGF levels were suppressed for at least 1 week after IVR. Only one case report found that the serum level of VEGF remained normalized until 4 weeks after IVR^4^. In another study, serum VEGF levels were significantly reduced in 9 patients after IVB and 5 patients after intravitreal aflibercept (IVA) up to 12 weeks, and the serum levels of VEGF were more suppressed in the IVB group than in the IVA group^22^.

However, serum VEGF concentrations varied broadly among different studies. Generally, systemic VEGF suppression seems to be more pronounced in IVB than IVR, but no studies have reported changes in serum VEGF concentration in ROP patients after IVC. In this study, we observed that serum concentrations of VEGF-A and VEGF-D were initially suppressed at 1 week after IVC and returned to baseline at 4 weeks. Conbercept is an engineered fusion protein with a molecular weight of 143 kDa that binds to all VEGF-A isoforms and the related VEGFR-1 ligands VEGF-B and PlGF. This drug has 50 times the VEGF binding affinity of bevacizumab or ranibizumab and washes out according to first-order kinetics in ocular tissues^23^^.^ Systemic VEGF suppression after intravitreal anti-VEGF drugs is associated with the drug dosage, drug-free intervals, half-life, molecular size and presence of Fc function^24^^.^ The vitreous half-life of conbercept is 4.2 days in rabbits, while those of aflibercept, bevacizumab and ranibizumab are 4.79 days, 6.61 days and 2.88 days, respectively^25–27^. The serum level of conbercept in 6 patients was undetectable within 1–2 days after injection in a previous study^28^. In our previous comparative study in adult patients with age-related macular degeneration^29^^,^ serum VEGF concentrations were significantly decreased at 1 day and 1 week after intravitreal injection of 0.5 mg conbercept, while no significant effect was observed at 4 weeks; however, in the IVR group, no significant difference was observed at any of the observed time points, indicating that conbercept might have a longer systemic VEGF suppression time than IVR. The same results were detected in ROP patients. Our previous study demonstrated that plasma VEGF was suppressed at 1 day after IVR and normalized at 1 week after injection^20^. In this study, the dose of conbercept was reduced to 0.25 mg per eye, which is half the adult dosage for ROP, and 57 patients received a total of 0.5 mg conbercept. Serum concentrations of VEGF-A and VEGF-D were initially suppressed 1 week after IVC and returned to baseline at 4 weeks. Three patients were injected in only one eye, and we were unable to statistically analyze whether this affected systemic VEGF levels. Considering the suggestion from our previous study that 0.15 mg IVC was effective for Zone II Stage 2/3 ROP with plus disease,^6^ reducing the doses of the anti-VEGF agents appears to be a safer choice that reduces serum VEGF suppression. Moreover, there was no difference in serum VEGF concentrations between ROP patients with recurrence and other patients before or after IVC treatment. Overall, this study found no prolonged systemic VEGF suppression after IVC in ROP patients.

In this study, recurrence that required treatment occurred in 9 patients (15 eyes, 12.82%) with ROP. Our previous studies^6,7^ of conbercept demonstrated similar ROP recurrence rates (6/38 and 3/20, respectively), which were consistent with a study from another team in South China (8/48).^8^ In our comparative study,^7^ the recurrence rate was lower with conbercept than with ranibizumab (15/28, 53.6%). Our previous study found a similar recurrence rate (45.45%) of ranibizumab.^20^ Recently, another study found that 26.2% (18/42) of eyes had a recurrence of ROP after the initial IVR treatment in South China.^21^ Overall, it seems that conbercept is associated with a lower clinical recurrence rate than ranibizumab. At the end of the follow-up, no ocular or systemic adverse events were observed in this group.

In addition, no significant differences were observed in the serum levels of other cytokines between baseline and 1 week or 4 weeks after IVC injection. Conbercept can bind to PlGF, and a previous study reported that retinal PlGF levels were decreased significantly in the conbercept (KH902)-treated group in an oxygen-induced retinopathy model.^30^ However, the serum level of PlGF was not suppressed in this study.

Furthermore, this study showed that the levels of several cytokines were significantly different between ROP patients and the control group, adding information on the pathogenesis of ROP. The key role of VEGF in the pathogenesis of ROP has been well illustrated in many animal models as well as in humans. Previous studies detected no difference in cord blood VEGF concentrations at birth between preterm and term infants,^31^ between preterm infants who later developed ROP and those who did not develop this disease in a gestational age-matched case–control study,^32^ or between ROP and non-ROP infants at 32 or 36 weeks.^33^ In this study, circulatory VEGF-A and VEGF-D concentrations in ROP patients were significantly higher than those in infants with congenital cataract. The pathogenesis of ROP is considered to involve two phases. Phase I is characterized by downregulation of growth factors, and phase II is marked by overproduction of VEGF, leading to vasoproliferation. In our study, the ROP patients were in phase II, and so their VEGF concentrations were higher than those of the control group, consistent with other studies.^34,35^

In addition, the serum levels of MIP-1β, IP-10, RANTES, eotaxin, TNF-α, and IL-18 were significantly higher in ROP patients than in controls, while the opposite was true of MCP-1 levels, suggesting that inflammation is involved in the development of ROP. MIP-1β, TNF-α and IL-18 are proinflammatory factors that can compromise the blood–retinal barrier, exacerbate retinal ischemia, modulate angiogenesis, and induce the progression of ROP. Chemotactic cytokines, such as RANTES, IP-10, and eotaxin, chemoattract various inflammatory cells such as natural killer cells and monocytes, stimulating inflammatory cascades in many diseases.^36,37^ MCP-1 has been detected in neurons and astrocytes and is involved in the development of the brain and neuroretina.^38^ In a gestational age-matched case–control study, the serum level of MIP-1β was significantly higher in ROP patients than in healthy preterm neonates and was negatively correlated with birthweight.^13^ Several studies observed high plasma levels of TNF-α in association with an increased risk of ROP,^14,15^ while BDNF levels were negatively correlated with ROP risk.^15^ A large cohort study suggested that RANTES and IL-18 might be involved in the pathogenesis of ROP.^12^ In another study, vitreous levels of IP-10, eotaxin, and RANTES were significantly higher in ROP eyes than in non-ROP eyes^39^. Elevated aqueous levels of eotaxin, IFN-γ, IP-10, MIP-1β, and TNF-α were detected in ROP patients, and MIP-1β levels were independently correlated with ROP retreatment.^40^ These data were consistent with our findings.

Moreover, serum levels of EGF, SCF, PlGF-1, and β-NGF were significantly higher in ROP patients than in controls in this study. A previous study demonstrated that EGF receptor inhibitors prevented insulin-induced retinal vascular leakage in mice,^41^ and inhibition of PlGF-1 reduced pathological vascular leakage in a mouse retinopathy model.^42^ SCF was observed to be upregulated by hypoxia, directing the migration of neural progenitor cells and promoting angiogenesis.^43^ NGF has been found in the adult and developing rodent retina in association with associated with retinal degeneration.^44^ The vitreous level of NGF was significantly elevated in diabetic retinopathy patients.^45^ The role of these cytokines in ROP has not been established, and further studies are needed to achieve a better understanding of the pathogenesis of ROP. In summary, it seems that cytokines are intricately interrelated with the development of ROP.

Our study has several limitations that must be mentioned. The sample size was small, and the serum levels of several cytokines varied widely. In addition, the serum concentration of VEGF did not necessarily reflect the levels in the retina,^46^ and it varied in different studies, probably as a result of different patient selection criteria, stages of ROP, samples, and test methods. Moreover, the control group of this study included full-term infants with congenital cataract, and there was a significant difference in age. Thus, the differences in cytokine levels between the two groups may have been interfered with by gestational age and postmenstrual age.

## Conclusion

In conclusion, IVC for ROP patients induced circulatory VEGF suppression at 1 week after injection, and this effect fully subsided at 4 weeks. IVC is an effective treatment for ROP patients, but its safety profile needs further investigation.

## Supplementary information

Supplementary information.

## Data Availability

The original data used to support the findings of this study are available from the corresponding author upon request.
