# Supplementary material for: Serum levels of cytokines in infants treated with conbercept for retinopathy of prematurity
Source: Sci Rep. 2020 Jul 29;10:12695. doi: 10.1038/s41598-020-69684-7 (PMC7391743; doi:10.1038/s41598-020-69684-7)
Supplement: Supplementary file 1 — Supplementary information. [file 41598_2020_69684_MOESM1_ESM.docx]

**Serum levels of cytokines in infants treated with conbercept for retinopathy of prematurity**

Yong Cheng^1*^, MD, Xuemei Zhu^1*^, MD,Dandan Linghu^1^, MD, Yongsheng Xu,^1,2^ MD, Jianhong Liang^1^, MD

**Supplemental Table 1**

**Serum concentrations (pg/ml) (mean ± SD) of cytokines in ROP patients after IVC treatment**

| Cytokines | baseline | 1 week | 4 weeks |
| --- | --- | --- | --- |
| VEGF-A | 1976.18±982.45 | 1388.99±753.27 | 1771.53±581.78 |
| VEGF-D | 110.23±99.57 | 87.88±90.34 | 111.06±79.99 |
| MIP-1β | 194.64±146.67 | 218.68±129.73 | 185.91±126.51 |
| Eotaxin | 29.72±24.13 | 38.70±23.60 | 42.15±18.67 |
| IP-10 | 136.99±111.45 | 136.11±67.47 | 164.26±59.40 |
| RANTES | 27.50±17.30 | 34.93±15.77 | 34.41±10.73 |
| IL-18 | 48.97±41.92 | 50.59±32.63 | 79.99±108.44 |
| TNF-α | 14.30±8.1 | 16.25±3.50 | 20.05±10.21 |
| MCP-1 | 232.96±255.37 | 237.90±144.54 | 220.25±145.41 |
| EGF | 50.31±39.13 | 63.72±31.03 | 74.71±23.10 |
| SCF | 23.50±15.91 | 25.10±15.86 | 23.66±17.94 |
| PIGF-1 | 45.62±37.48 | 59.80±38.81 | 69.12±33.78 |
| β-NGF | 22.31±16.00 | 26.51±15.51 | 25.93±14.34 |
| HGF | 113.47±113.59 | 155.18±116.16 | 104.09±85.90 |
| SDF-1α | 268.55±204.31 | 297.06±123.05 | 349.66±197.88 |
| GRO-α | 28.26±35.3 | 32.12±30.3 | 23.88±12.73 |
| BDNF | 62.75±78.73 | 95.29±112.98 | 59.39±32.68 |
| IFN-γ | 58.74±59.31 | 46.06±22.24 | 35.74±23.25 |
| IL-8 | 16.32±29.74 | 9.20±9.81 | 10.64±8.23 |
| IL-6 | 14.31±10.50 | 11.19±6.21 | 13.56±8.81 |
| MIP-1α | 17.26±23.20 | 15.40±8.08 | 7.60±6.94 |
| IL-1RA | 892.19±1388.95 | 688.92±503.07 | 634.19±357.08 |
| FGF-2 | 16.63±8.73 | 16.56±5.25 | 20.80±9.58 |
| PDGF-BB | 243.53±388.90 | 217.84±186.23 | 283.15±239.77 |

**Notes:** Data are shown as mean ± SD.
